# Effects of Hypergravity on Osteopontin Expression in Osteoblasts

**DOI:** 10.1371/journal.pone.0128846

**Published:** 2015-06-05

**Authors:** Shuai Zhou, Yan Zu, Zhenglong Sun, Fengyuan Zhuang, Chun Yang

**Affiliations:** 1 School of Biological Science and Medical Engineering, Beihang University, Beijing, China; 2 Institute of Biomechanics and Medical Engineering, School of Aerospace Engineering, Tsinghua University, Beijing, China; The Forsyth Institute, UNITED STATES

## Abstract

Mechanical stimuli play crucial roles in bone remodeling and resorption. Osteopontin (OPN), a marker for osteoblasts, is important in cell communication and matrix mineralization, and is known to function during mechanotransduction. Hypergravity is a convenient approach to forge mechanical stimuli on cells. It has positive effects on certain markers of osteoblast maturation, making it a possible strategy for bone tissue engineering. We investigated the effects of hypergravity on OPN expression and cell signaling in osteoblasts. Hypergravity treatment at 20 g for 24 hours upregulated OPN expression in MC3T3-E1 cells at the protein as well as mRNA level. Hypergravity promoted OPN expression by facilitating focal adhesion assembly, strengthening actin bundles, and increasing Runx2 expression. In the hypergravity-triggered OPN expression pathway, focal adhesion assembly-associated FAK phosphorylation was upstream of actin bundle assembly.

## Introduction

Mechanical stimuli play important roles in bone remodeling. Bone cells differentiate, proliferate, and mature under mechanical stress *in vivo*, and are extremely sensitive to mechanical cues [1–5]. Engineered bone tissue cultured *in vitro* always fails to provide adequate tissue structure and mechanical properties. This is largely attributed to the lack of mechanical stimuli during cell culture. Hypergravity, or centrifugation, is a convenient approach to place mechanical stress on cultured cells, and is known to have an impact on osteogenesis [6]. In previous studies, low-level (<10 ×*g*), mid-level (10–100 ×*g*), and high-level (>100 ×*g*) hypergravity conditions were all observed to have significant effects on the morphology, proliferation, gene transcription, extracellular matrix protein synthesis, and alkaline phosphatase activity of osteoblasts. These findings suggested that hypergravity is a possible approach for bone tissue engineering [7–10]. To evaluate the impact of hypergravity on osteogenesis, a detailed analysis of its effects on osteoblasts should be conducted and the mechanisms underlying osteoblast response should be explored.

During bone regeneration and development, OPN, a marker for osteoblasts, plays an important role in cell communication and matrix mineralization [11–16]. Recently, it was found that OPN is regulated by mechanical forces, and contributes to the mechanotransduction pathway. Shear stress, mechanical stretch, and water pressure are all known to affect OPN expression in a variety of cell types, such as osteoblasts, osteocytes, and bone marrow mesenchymal stem cells [17–21]. Gravity was also shown to have a significant impact on OPN expression in human periodontal ligament fibroblasts, MG-63 osteoblasts, endothelial cells, and ML-1 cells [22–25]. Ishijima et al. also showed that OPN was responsible for bone loss in the tail suspension mouse model that is often used to mimic microgravity effects on lower limbs *in vivo* on earth [26–28]. However, the mechanism underlying the change in OPN expression in cells due to altered gravity warrants further investigation.

In the present study, we aimed to understand the mechanism by which hypergravity affects OPN expression in osteoblasts. Using a custom-designed centrifuge, we cultured cells under 20-g hypergravity conditions, and found that OPN expression and secretion were upregulated in MC3T3-E1 osteoblast cells. Runx2, a major regulator of osteogenic differentiation, was required for hypergravity-induced OPN expression. A multi-index quantification showed that the cytoskeleton and focal adhesions of MC3T3-E1 cells were altered by hypergravity, and they also contributed to hypergravity-induced OPN expression. Taken together, our findings showed that hypergravity may affect OPN expression via changes in focal adhesions, the cytoskeleton, and Runx2.

## Materials and Methods

### Mechanical device

**Fig 1:**
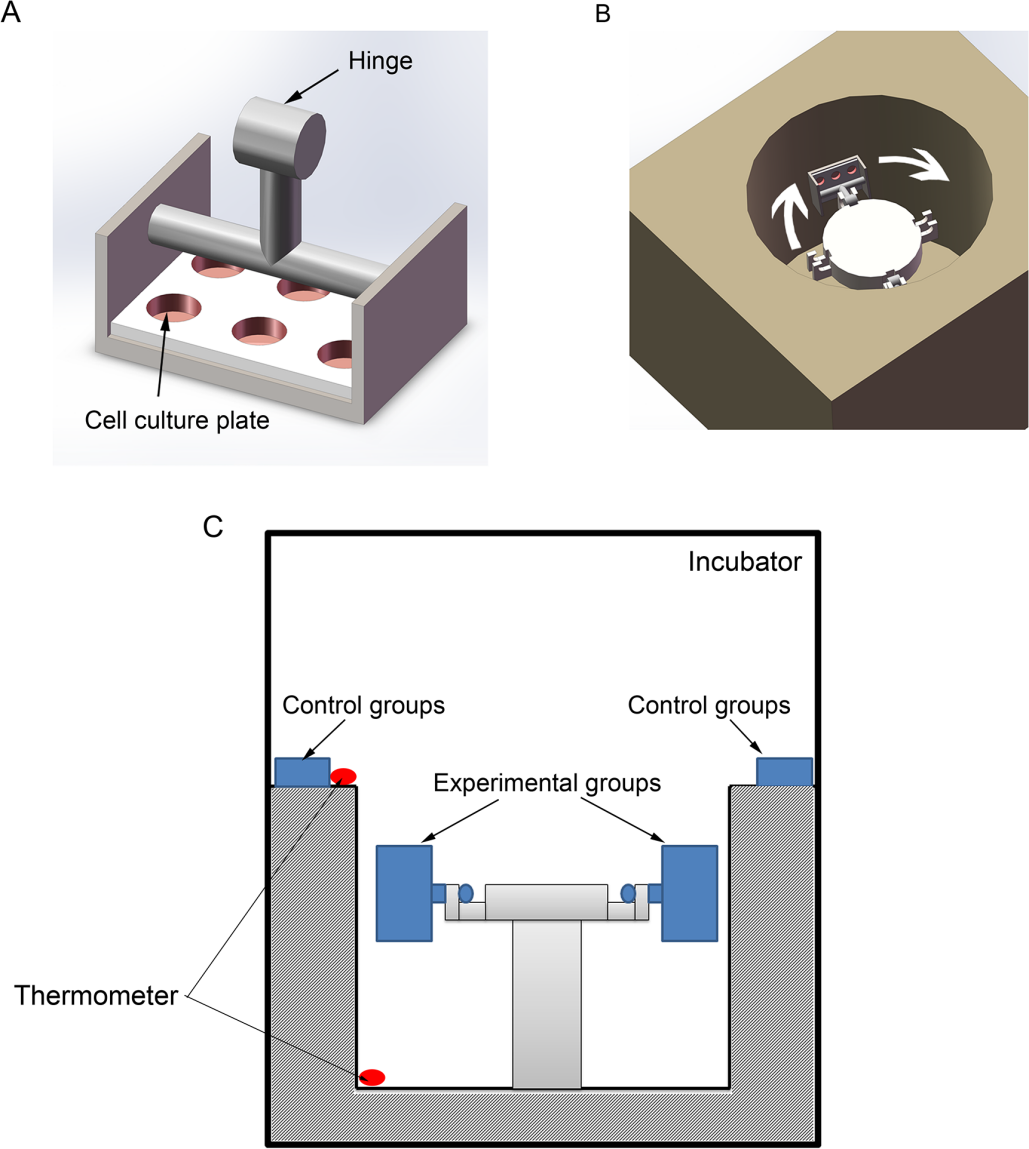
The mechanical hypergravity device. (A) Two horizontal rotors were made to fit the 6-well culture plates. Each rotor can also carry 2 culture flasks or 6 separated culture dishes. (B) Isometric view of the centrifuge. (C) A diagram showed the experimental setup. The control cells were placed on the top of the centrifuge and the hypergravity-treated cells were put in the rotors of the centrifuge. The centrifuge was placed in an incubator at 37°C. Thermometers were used to measure the temperature inside the centrifuge and near the control cells.

A custom-made low-speed centrifuge was used to apply hypergravity to the cells in this study. Cells in culture flasks (or dishes) were placed on the horizontal rotors in the centrifuge, and 20 ×*g* was applied for 24 h in an incubator at 37°C. The control cells were placed on the platform of the centrifuge, as shown in Fig 1. The temperature was measured on the platform, where the control cells were placed, and in the centrifuge. The temperature was measured by THG312 thermometer (Mettler Toledo, Switzerland), and the value was 36.7 ± 0.2°C at the platform, and 36.8 ± 0.3°C in the incubator.

### Cell culture

MC3T3-E1 osteoblastic cells (Cell Center; School of Basic Medicine of Peking Union Medical College) were purchased at the passage number of 20, and were maintained in Dulbecco’s Modified Eagle Medium (DMEM) supplemented with 10% fetal bovine serum (FBS), 2 mmol/L L-glutamine, and 1% penicillin–streptomycin at 37°C in 5% CO_2_. Cells were sub-cultured (1:3), every 3 days, using 0.25% trypsin with 1 mM EDTA. 15mM Hepes was added to adjust the pH in culture medium. Unless otherwise specified, 10^5^ cells per flask were exposed to 20 ×*g* hypergravity for 24 h, after seeding and spreading for 2 h. The control cells were subjected to the same conditions as the hypergravity-exposed cells in terms of timing, incubation media, and other procedures. The MC3T3-E1 cells used in this study were from passages 25 to passages 30. We immediately collected n = 6 1 ×*g* controls and n = 6 20 ×*g* hypergravity treated samples for western blotting analyses as well as n = 6 1 ×*g* controls and n = 6 20 ×*g* hypergravity treated samples for real-time PCR, respectively.

In some experiments, groups of cells were placed in incubator for a period of time after 1 ×*g* and 20 ×*g* treatments; and samples were collected at 25 h, 26 h, and 27 h. We collected n = 6 1 ×*g* controls and n = 6 20 ×*g* hypergravity treated samples from each class. In experiments with varies cell densities, 10^3^, 10^4^, 10^5^, or 10^6^ cells were exposed to 20 ×*g* hypergravity for 24 h. In experiments with altered preparatory adherent period, cells were preparatory adhered over a range of 6 h, 12 h, and 24 h and then exposed to 20 ×*g* hypergravity for 24 h. We collected n = 6 1 ×*g* controls and n = 6 20 ×*g* hypergravity treated samples from each class. In addition, cells were exposed to 20 ×*g* hypergravity for a longer period of 48 h or 72 h. We then collected n = 4 1 ×*g* controls and n = 4 20 ×*g* hypergravity treated samples from each condition for western blotting analyses.

In blebbistatin (Sigma, St. Louis, MO) treatment experiments, MC3T3-E1 cells were treated with 50 μM blebbistatin for 24 h during 20 ×*g* hypergravity stimulation (n = 6). In FAK inhibitor (PF573228; Sigma, St. Louis, MO) experiments, cells were treated with 10 mM PF573228 for 24 h during 20 ×*g* hypergravity stimulation. We collected n = 6 1 ×*g* controls and n = 6 20 ×*g* hypergravity treated samples for western blotting analyses as well as n = 4 1 ×*g* controls and n = 4 20 ×*g* hypergravity treated samples for real-time PCR, respectively. To measure the secreted OPN in the culture medium, MC3T3-E1 cells were first seeded on culture flasks with complete media. After adhering and spreading, cells were cultured in serum-free media and then exposed to 20 ×*g* hypergravity for 24 h. The culture media were collected and concentrated using Millipore’s Amicon Ultra 15 mL centrifugal filter device, according to the manufacturer’s protocol. We collected 1 ×*g* controls and 20 ×*g* hypergravity treated samples without drug (n = 9), with blebbistatin (n = 6) or with PF573228 (n = 5) for western blotting analyses.

The primary osteoblasts were obtained from 3-day-old mouse calvarias following the published method [29, 30]. In brief, the skulls were dissected and the periosteum and endosteum were stripped off. Then the bone was cut into pieces and digested with 2.5 mg/ml typsin for 30 min and 1.0mg/ml collagenase II twice for 1h. The cells were collected and cultured in α-MEM supplemented with 10% fetal bovine serum (FBS), 2 mmol/L L-glutamine, and 1% penicillin–streptomycin at 37°C in 5% CO_2_. The osteoblasts from passages 2 to passages 6 were used in this study. We collected n = 6 1 ×*g* controls and n = 6 20 ×*g* hypergravity treated samples for western blotting analyses.

### Western blotting analysis

Whole cell lysates from control (1 ×*g*) and hypergravity-exposed (20 ×*g*) cells were prepared with RIPA lysis buffer. Protein content was determined using a Protein Assay Kit (Thermo). Equal amounts of protein samples were resolved by reducing 10% SDS-PAGE and transferred to a PVDF membrane. The membrane was blocked for 2 h in 5% nonfat milk and incubated with primary antibodies against OPN (1:1000, Abcam), Runx2 (1:1000, Abcam), Paxillin (1:1000, Santa Cruz Biotechnology), p-FAK (1:1000, Cell Signaling), FAK (1:500, Santa Cruz Biotechnology), and GAPDH (1:2000, Santa Cruz Biotechnology) overnight at 4°C. The membranes were then incubated with horseradish peroxidase-conjugated anti-mouse IgG (1:10,000) or anti-rabbit IgG (1:10,000) for 2 h at room temperature. Lastly, the membranes were developed with enhanced chemiluminescence reagents (Millipore) and exposed to an X-ray film (Eastman-Kodak, Rochester, NY, USA).

### siRNA Transfection

Mouse Runx2 siRNA and control siRNA were transfected using Neon Transfection System (Invitrogen), according to the manufacturer’s instructions. The Runx2 siRNA sequences (sense, 5′-CAGACAAGUGAAGAGGUUUU-3′; antisense, 5′-AACCUCUUCACUUGUCUGUU-3′) were used in a previous study [31]. After 8-h transfection, cells were subjected to 20 ×*g* hypergravity for 24 h and processed for OPN and Runx2 expression analysis. We collected n = 6 1 ×*g* controls and n = 6 20 ×*g* hypergravity treated samples for western blotting analyses.

### Quantitative real-time PCR

Total RNA was extracted from cell samples using TRIzol (Invitrogen, Carlsbad, CA, USA), and cDNA was generated using a reverse transcription kit (Tiangen), according to the manufacturer’s instructions. SYBR Green Realtime PCR Master Mix (TOYOBO) was used for the real-time PCR analysis. The reactions were performed in a Mastercycler ep realplex (Eppendorf, Germany). The following primers were designed using the Primer 3 website and were synthesized by Invitrogen:

*OPN* (sense, 5′-TGCACCCAGATCCTATAGCC-3′;

antisense, 5′-CTCCATCGTCATCATCATCG-3′),

*Runx2*, (sense, 5′-CCGAAATGCCTCCGCTGTTATG-3′;

antisense, 5′-GGATTTGTGAAGACTGTTATGGT-3′),

GAPDH, (sense, 5′-TGCACCACCAACTGCTTAG-3′;

antisense, 5′-GGATGCAGGGATGATGTTC-3′).

A comparative CT method was used for quantification. The housekeeping gene GAPDH was used for internal normalization.

### Immunofluorescence staining

Cells were washed with PBS, fixed with 4% paraformaldehyde for 10 min, and permeabilized in 0.1% Triton X-100 buffer for 5 min. After blocking in 5% BSA for 30 min at room temperature, the cells were incubated with primary antibodies against OPN (1:500, Abcam), Runx2 (1:200, Abcam), and paxillin (1:200, Santa Cruz) diluted in 5% BSA at 4°C overnight. Cells were then incubated with Anti-mouse IgG conjugated to TRITC (1:200, Abcam), anti-rabbit IgG conjugated to FITC (1:200, Abcam), and rhodamine phalloidin (1:100, Abcam) for 2 h at room temperature. Lastly, cell nuclei were visualized by DAPI (Sigma) and viewed under a Leica TCS SP5 confocal microscope system. The fluorescence images were selected randomly for quantification. The quantification of the fluorescence signal was done using the image processing software Image-Pro plus (version 6.0, Media Cybernetics, LP) and the ImageJ software. To measure the thickness of actin bundles, we drew a line across each cell, and the bundle width at the cross line section was quantified. To avoid background noise, only regions with a fluorescence intensity higher than 40 were considered as actin bundles and thus counted.

### Statistical analysis

All experiments in this study were performed at least quadruplicate. The Wilcoxon matched pairs test and Kruskal–Wallis test were carried out to compare the differences between the experimental groups in realtime-PCR and western blotting. Unpaired t-test was used to compare the differences between the two experimental groups in immunofluorescence staining. A *P*-value < 0.05 was considered significant.

## Results

### Hypergravity increases OPN and Runx2 expression

**Fig 2:**
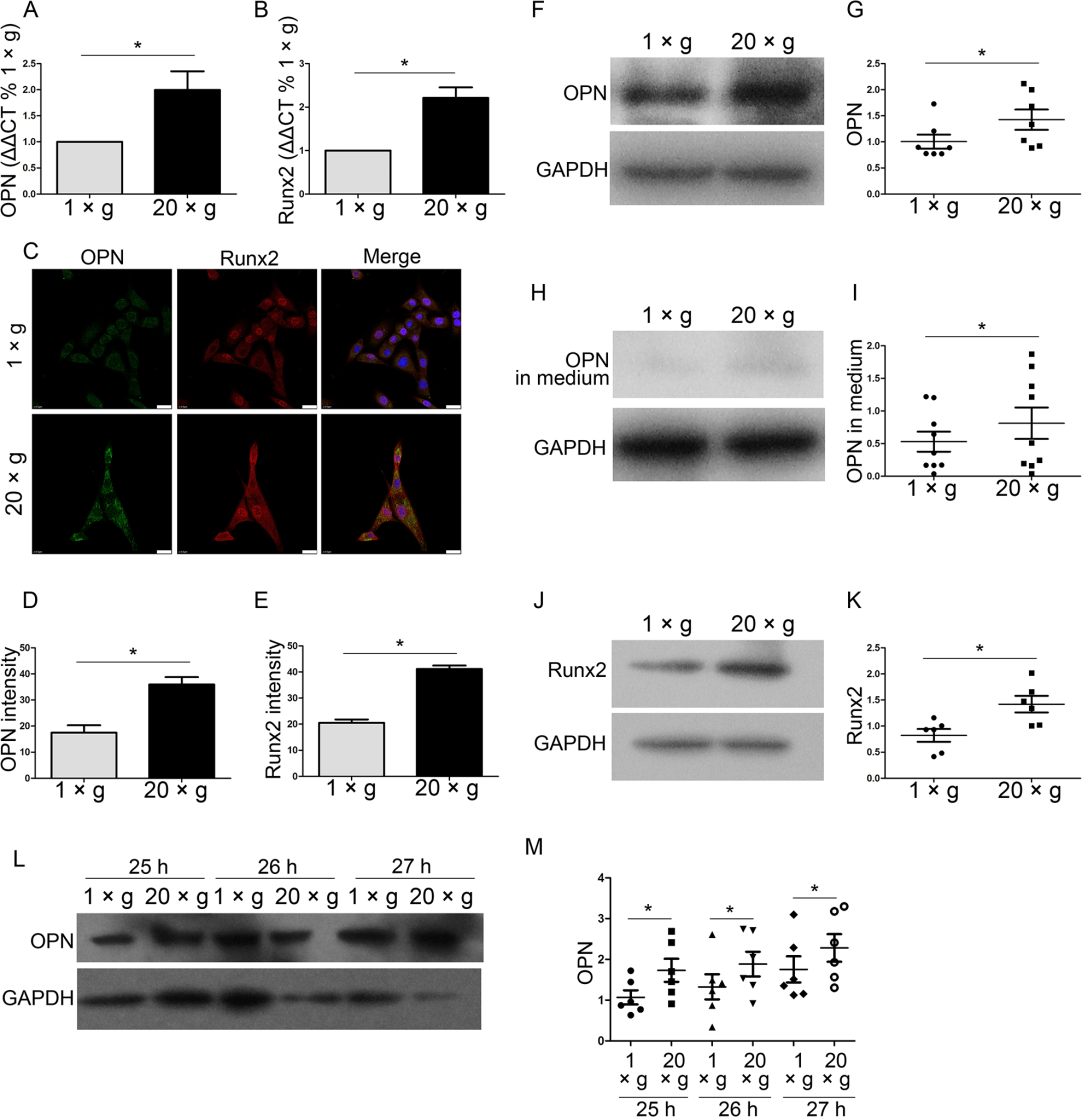
Hypergravity increased the expression of OPN and Runx2. The mRNA and protein expression levels of OPN and Runx2 in MC3T3-E1 cells after 24 h at either 1 ×*g* or 20 ×*g* are shown. (A) Comparative CT quantitation of real-time PCR results of OPN expression (mean ± SEM; n = 6). (B) Comparative CT quantitation of real-time PCR results of Runx2 expression (mean ± SEM; n = 6). (C) Immunofluorescence staining of cells under 1 ×*g* or 20 ×*g* hypergravity. Panels show individual immunostains for the OPN, Runx2, and an overlay of the two with nuclei. OPN, Runx2 and nuclei were visualized by FITC (green), TRITC (red) and DAPI (blue) (Scale bar = 25 μm). (D) The mean fluorescence intensity of OPN. ~50 cells were counted (mean ± SEM). (E) The mean fluorescence intensity of Runx2. ~50 cells were counted (mean ± SEM). (F and G) Western blotting results of OPN expression in cells and statistical analyses (n = 7). (H and I) Western blotting results of secreted OPN in medium and statistical analyses (n = 9). (J and K) Western blotting results of Runx2 expression in cells and statistical analyses (n = 6). (L and M) Cells were exposed to 1 ×*g* or 20 ×*g* for 24 h, and then incubated at 37°C. The expression of OPN was measured at 25 h, 26 h, and 27 h by western blotting. Western blotting results of OPN expression in cells and statistical analyses were shown. (n = 6) n.s. indicates no statistical difference between 1 ×*g* and 20 ×*g*. *, statistically significant difference (*P* < 0.05).

OPN and Runx2 are typical markers of osteoblasts maturation and differentiation. To investigate whether hypergravity affects OPN and Runx2 expression, we exposed MC3T3-E1 cells, a well-established murine osteoblastic cell model used in bone studies, to 20 ×*g* centrifugal force for 24 h. After exposure to hypergravity, cells were assayed for the mRNA and protein levels of OPN and Runx2 by real-time PCR, immunofluorescence staining, and western blotting, respectively (Fig 2A–2K). We observed that hypergravity promoted an increased expression of OPN at both the mRNA (2-fold of control) (n = 6, p<0.05) and protein level (1.6–1.8-fold of control) (n = 7, p<0.05) (Fig 2A, 2C, 2D, 2F and 2G). Secreted OPN in the culture medium was also upregulated (1.4-fold of control) after hypergravity treatment (n = 9, p<0.05) (Fig 2H and 2I). The effect of hypergravity on OPN expression lasted for at least 3 hrs after the hypergravity exposure (n = 6, p<0.05) (Fig 2L and 2M). We also detected a 0.7- and 1.0-fold increase in Runx2 expression following hypergravity by immunofluorescence staining and western blotting (n = 6, p<0.05) (Fig 2C, 2E, 2J and 2K). Runx2 mRNA increased 1.2-fold after hypergravity treatment (n = 6, p<0.05) (Fig 2B). These results indicate that hypergravity at 20 ×*g* increases the expression and secretion of OPN, and the expression of Runx2.

**Fig 3:**
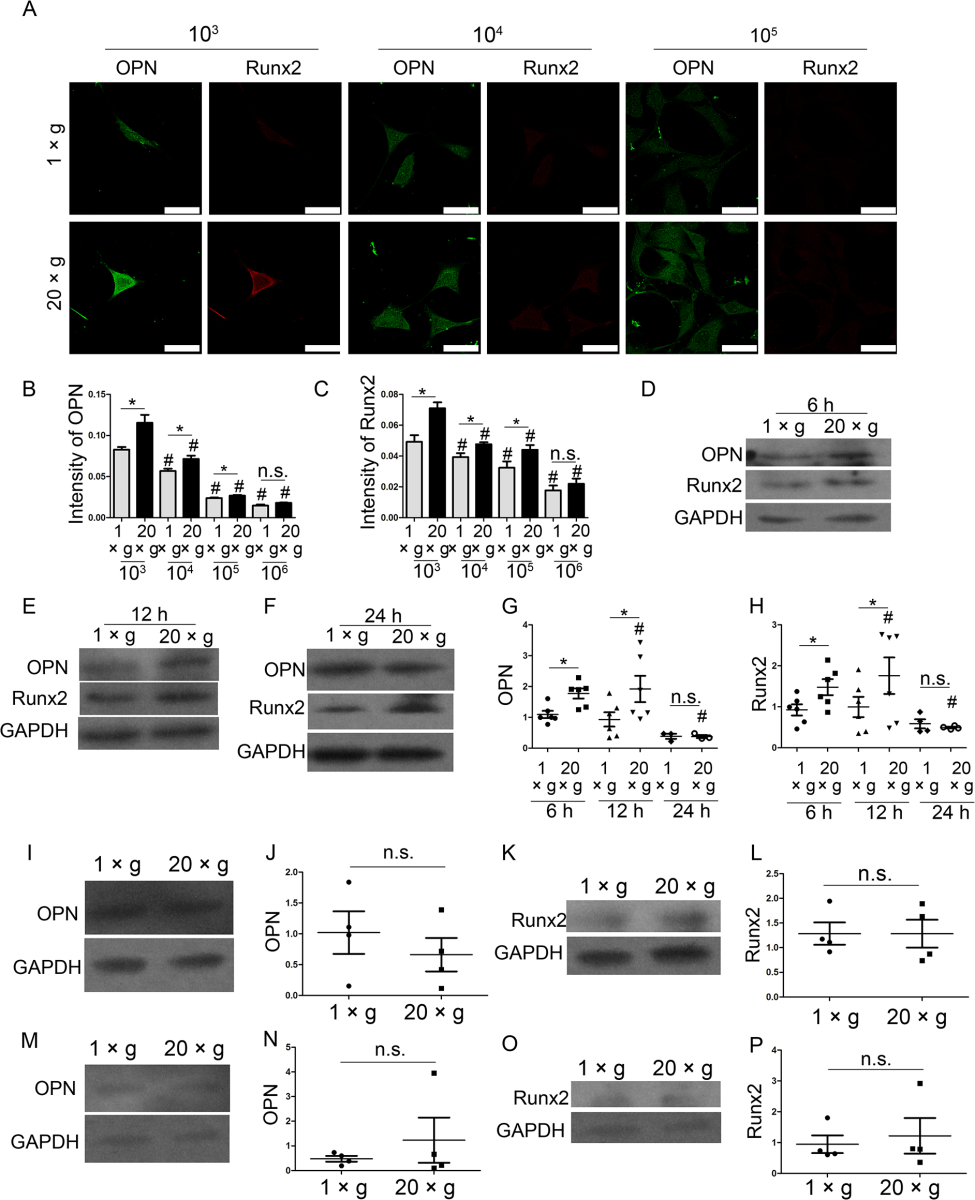
The hypergravity-increased OPN and Runx2 expression in MC3T3-E1 cells were affected by the cell density, the preparatory adherent period and the time length for hypergravity treatment. (A) Cells were seeded at the density of 10^3^, 10^4^, 10^5^, and 10^6^ and exposed to hypergravity. Immunofluorescence staining of cells at the density of 10^3^, 10^4^, and 10^5^ under 1 ×*g* or 20 ×*g* hypergravity was shown. OPN and Runx2 were visualized by FITC (green) and TRITC (red) (Scale bar = 50 μm). (B) The mean fluorescence intensity of OPN at diverse cell densities was shown. The counted cell numbers of each class were more than 50 (mean ± SEM). (C) The mean fluorescence intensity of Runx2 at diverse cell densities was shown. The counted cell numbers of each class were more than 50 (mean ± SEM). (D, E and F) The expression of OPN and Runx2 in cells under 1 ×*g* or 20 ×*g* with the preparatory adherent period of 6 h (D), 12 h (E) and 24 h (F) were shown. (G and H) Statistical analyses of OPN (G) and Runx2 (H) expression over the diverse preparatory period (n = 6). (I and J) Western blotting results of OPN expression in cells under 1 ×*g* and 20 ×*g* for 48 h and statistical analyses (n = 4). (K and L) Western blotting results of Runx2 expression in cells under 1 ×*g* and 20 ×*g* for 48 h and statistical analyses (n = 4). (M and N) Western blotting results of OPN expression in cells under 1 ×*g* and 20 ×*g* for 72 h and statistical analyses (n = 4). (O and P) Western blotting results of Runx2 expression in cells under 1 ×*g* and 20 ×*g* for 72 h and statistical analyses (n = 4). n.s. indicates no statistical difference between 1 ×*g* and 20 ×*g*. *, statistically significant difference (*P* < 0.05). #, statistically significant difference between the varies cell density classes or the 6 h, 12 h, and 24 h preparatory adherent period classes (*P* < 0.05) (either control or hypergravity groups).

For better understanding the effect of hypergravity on oesteoblasts, we assayed the effect of the time length for the gravity treatment, the preparatory adherent period before the hypergravity exposure, and cell density on hypergravity-induced increment of OPN and Runx2. To investigate the effect of cell density on hypergravity-increased OPN and Runx2 expression, the cells were seeded at the density of 10^3^, 10^4^, 10^5^, and 10^6^ and exposed to hypergravity. By immunofluorescence staining, we observed that at the cell density of 10^3^, 10^4^, 10^5^, hypergravity up-regulated the expression levels of OPN and Runx2 (Fig 3A–3C). At the density of 10^6^, OPN and Runx2 expression were all suppressed in both control group and hypergravity-treated group; and no difference was observed between these two groups (Fig 3B and 3C). We also examined the effect of different preparatory adherent period over a range of 6 h, 12 h, and 24 h, and found that OPN and Runx2 expression were up-regulated after hypergravity treatment at 6 h (n = 6, p<0.05) (Fig 3D, 3G and 3H), and 12 h pre-adhesion groups (n = 6, p<0.05) (Fig 3E, 3G and 3H). However, when plating cells on the flask for 24 h before the hypergravity exposure, the difference of OPN and Runx2 expression between hypergravity groups and the control groups disappeared (n = 6, p>0.05) (Fig 3F, 3G and 3H). MC3T3-E1 cells exposed to hypergravity for 48 and 72 hours showed no difference in the level of OPN and Runx2 between the control and hypergravity-treated groups (n = 4, p>0.05) (Fig 3I–3P).

**Fig 4:**
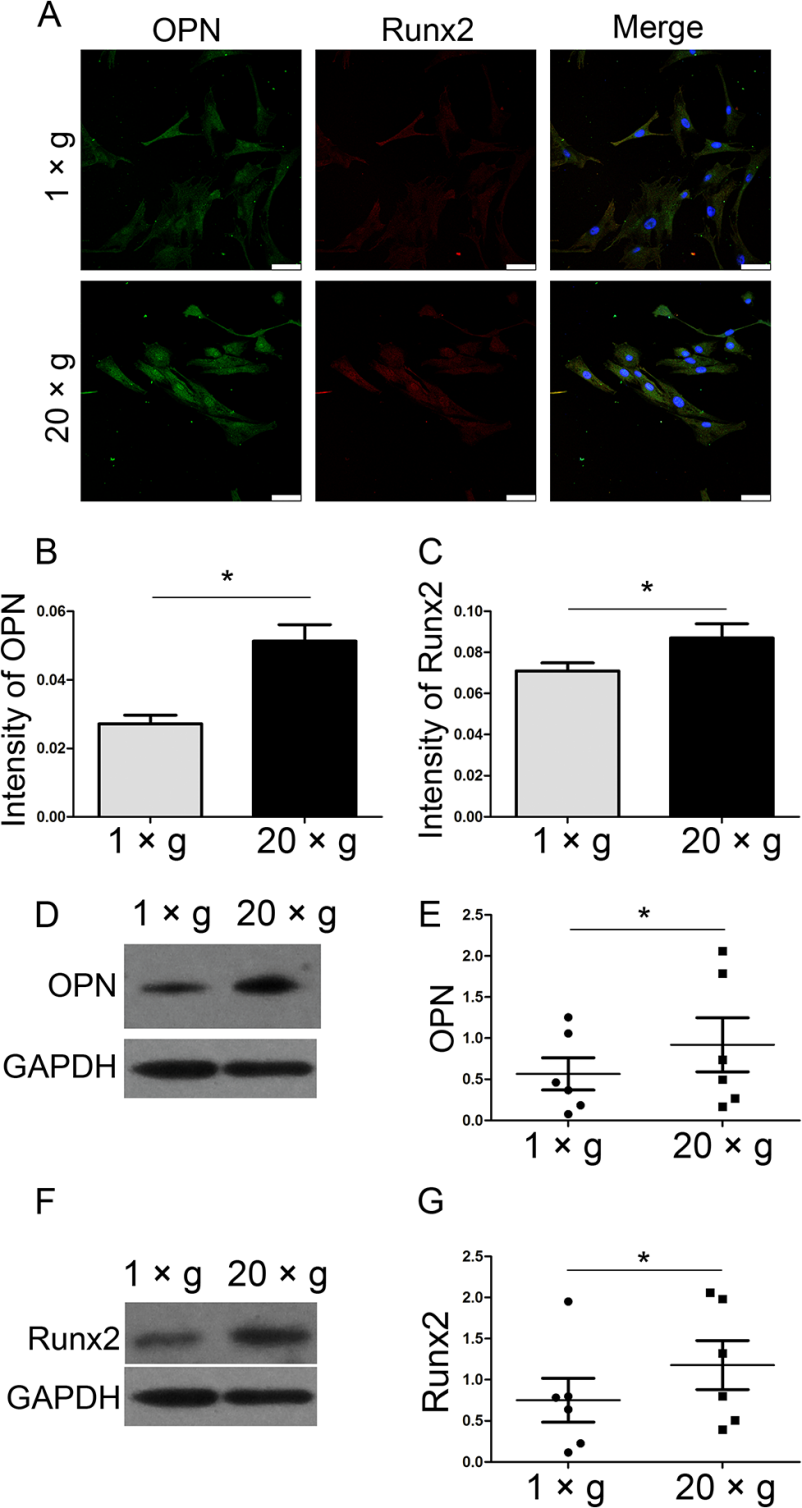
Hypergravity increased the expression of OPN and Runx2 in primary osteoblasts. (A) Immunofluorescence staining of primary osteoblasts under 1 ×*g* or 20 ×*g* for 24 h was shown. OPN, Runx2 and nuclei were visualized by FITC (green), TRITC (red) and DAPI (blue) (Scale bar = 50 μm). (B) The mean fluorescence intensity of OPN. ~50 cells were counted (mean ± SEM). (C) The mean fluorescence intensity of Runx2. ~50 cells were counted (mean ± SEM). (D and E) Western blotting results of OPN expression in primary osteoblasts under 1 ×*g* and 20 ×*g* for 24 h and statistical analyses (n = 6). (F and G) Western blotting results of Runx2 expression in cells under 1 ×*g* and 20 ×*g* for 72 h and statistical analyses (n = 6). n.s. indicates no statistical difference between 1 ×*g* and 20 ×*g*. *, statistically significant difference (*P* < 0.05).

To test the effect of hypergravity on OPN and Runx2 expression in primary osteoblast, we exposed murine calvarial osteoblasts to 20 ×*g* centrifugal force for 24 h. By immunofluorescence staining, we detected a 0.88- and 0.4-fold increase in OPN and Runx2 expression following hypergravity, respectively (Fig 4A–4C). Moreover, hypergravity-increased expression of OPN and Runx2 in primary osteoblasts were confirmed by western blotting (n = 6, p<0.05) (Fig 4D–4G).

Since OPN plays crucial roles in bone development, regeneration, and mechanotransduction, we next adopt MC3T3 as a model to explore how hypergravity affects OPN expression.

### Runx2 is upstream of hypergravity-regulated OPN expression

**Fig 5:**
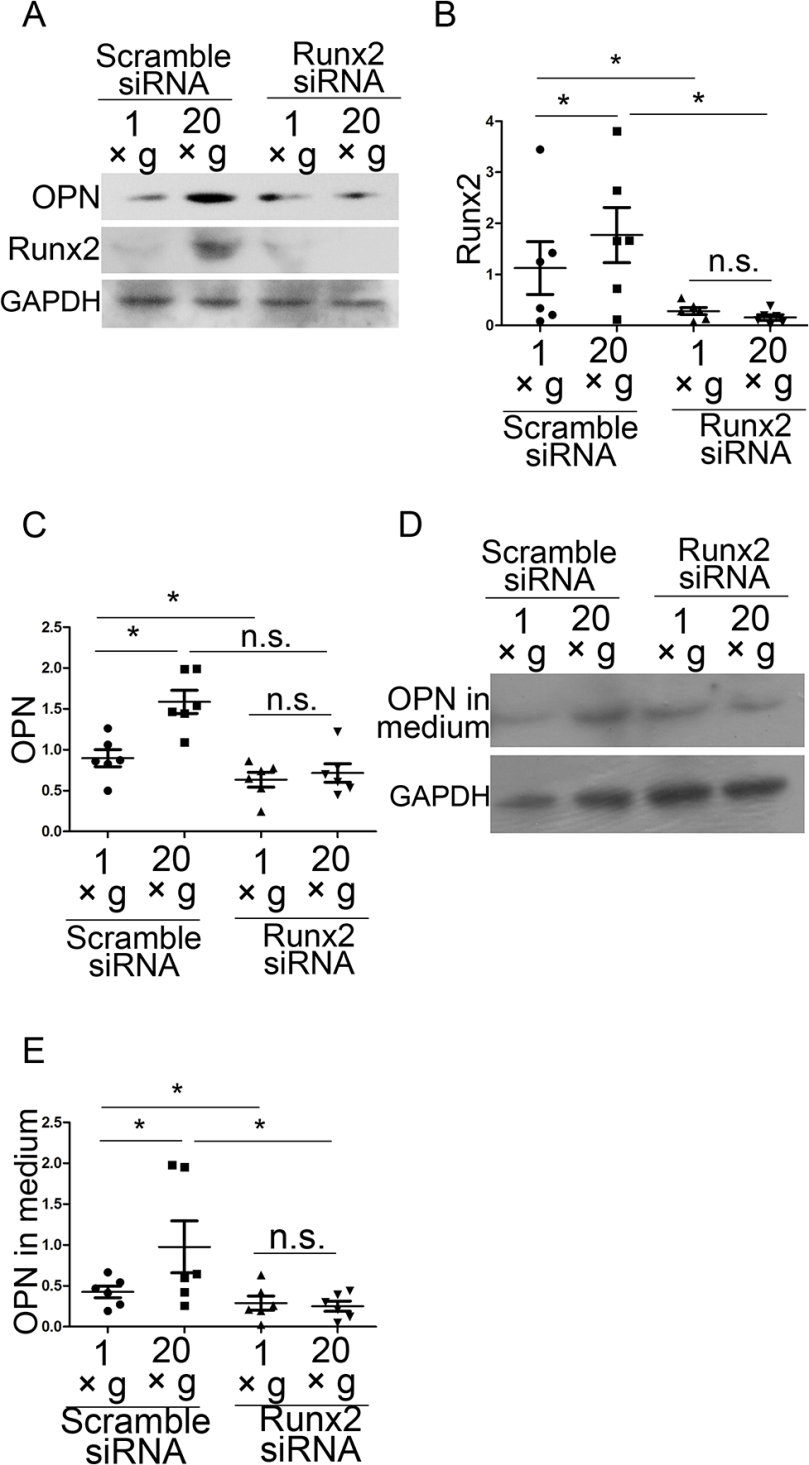
Hypergravity regulated OPN expression by promoting Runx2 expression. (A) Runx2 siRNA downregulated both Runx2 and OPN expression. (B and C) Statistical analyses of results in A (n = 6). (D) Western blotting results of OPN in culture medium after Runx2 siRNA transfection. (E) Statistical analyses of results in I (n = 6). n.s. indicates no statistical difference between 1 ×*g* and 20 ×*g*. *, statistically significant difference (*P* < 0.05).

Runx2, an indispensable transcription factor in the osteoblastic lineage, is required for OPN promoter activity, and it is associated with the differentiation of osteoblasts [32–35]. However, whether Runx2 plays a role in hypergravity-induced OPN expression is unclear. To examine the role of Runx2 in hypergravity-induced OPN expression, we transfected the cells with Runx2 siRNA and exposed them to 20 ×*g* hypergravity for 24 h. Runx2 knockdown blocked the differences in OPN expression and secretion between the 20 ×*g* hypergravity-treated and control groups, and downregulated OPN expression in both the groups (n = 6, p<0.05) (Fig 5). These results indicate that Runx2 is involved in the hypergravity-regulated OPN expression pathway. We next explored the upstream factors regulating OPN and Runx2 expression.

### Hypergravity strengthens the cytoskeletal system and focal adhesions in MC3T3-E1 cells

**Fig 6:**
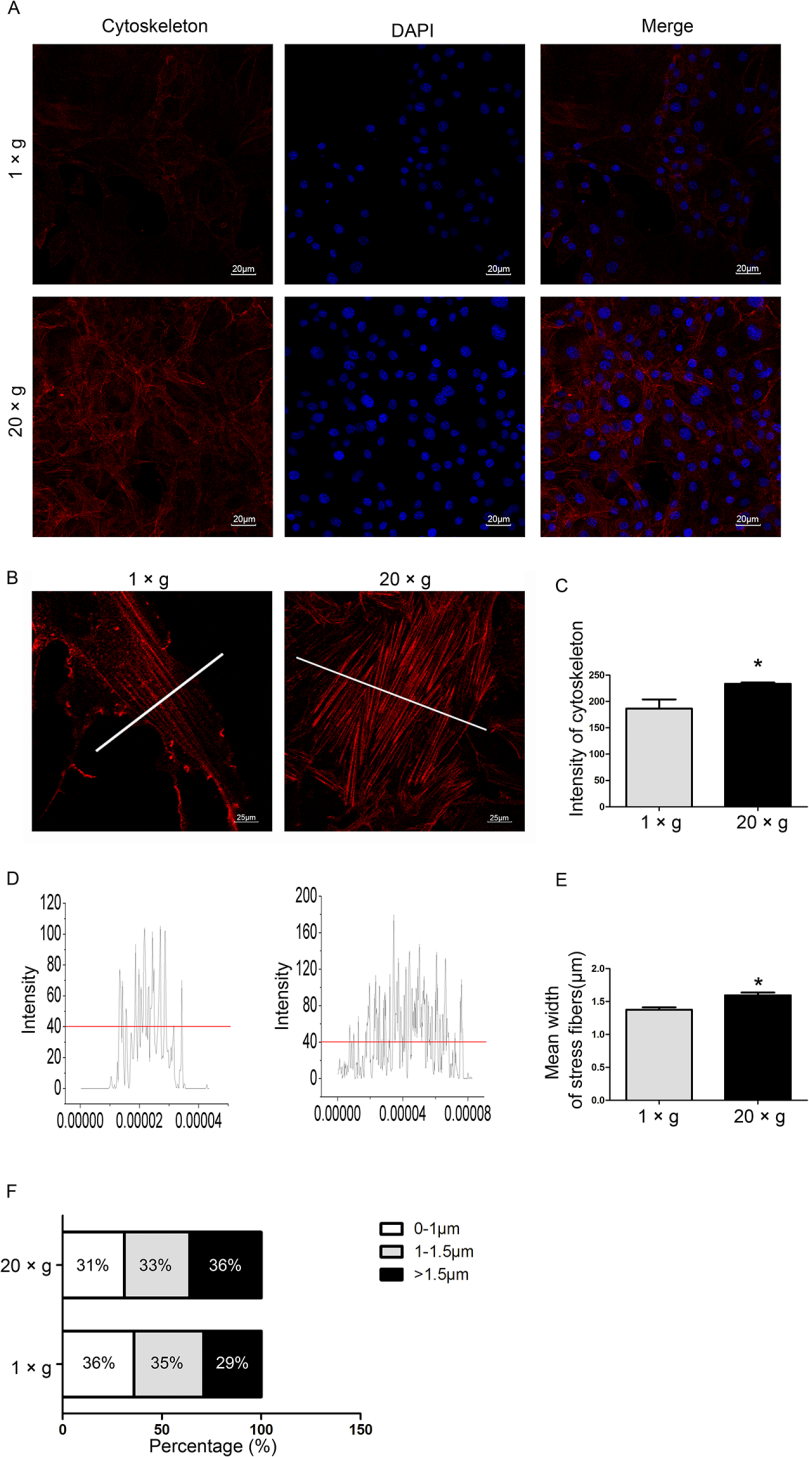
Effects of hypergravity on the cytoskeleton. (A) Rhodamine phalloidin (red) labeled the actin fibers and DAPI (blue) labeled the nuclei (Scale bar = 20 μm). (B) Enlarged view of actin bundles (red) (Scale bar = 25 μm). (C) The mean fluorescence intensity of the cytoskeleton. ~250 cells were counted (mean ± SEM). (D) The fluorescence distribution at the section where white lines are shown in B. (E) The mean width of actin bundles. ~200 filaments were counted (mean ± SEM). (F) The distribution of the width of actin bundles. n.s. indicates no statistical difference between 1 ×*g* and 20 ×*g*. *, statistically significant difference (*P* < 0.05).

The cytoskeletal system and focal adhesions (FAs) are known to be the mechanosensing elements in several mechanotransduction pathways [36–38]. Previous reports have demonstrated that the fluorescence intensity of labeled actin bundles increased in certain types of hypergravity-treated cells [25, 39]. However, not only the intensity, but also the distribution and thickness of actin bundles are crucial in characterizing the effects of hypergravity on cells. Using an integrative approach, we introduced a multi-index assay to characterize the impact of hypergravity on actin fibers and FAs. We observed that compared with unloaded cells, 20 ×*g* hypergravity-treated cells had higher actin staining intensity and thicker actin filaments. The average thickness of actin bundles was 1.39 μm in unloaded cells and 1.6 μm in the centrifuged cells. In addition, 36% of all visualized fibers in the loaded cells were thicker than 1.5 μm, compared with those in unloaded cells (29% of all visualized fibers) (Fig 6).

**Fig 7:**
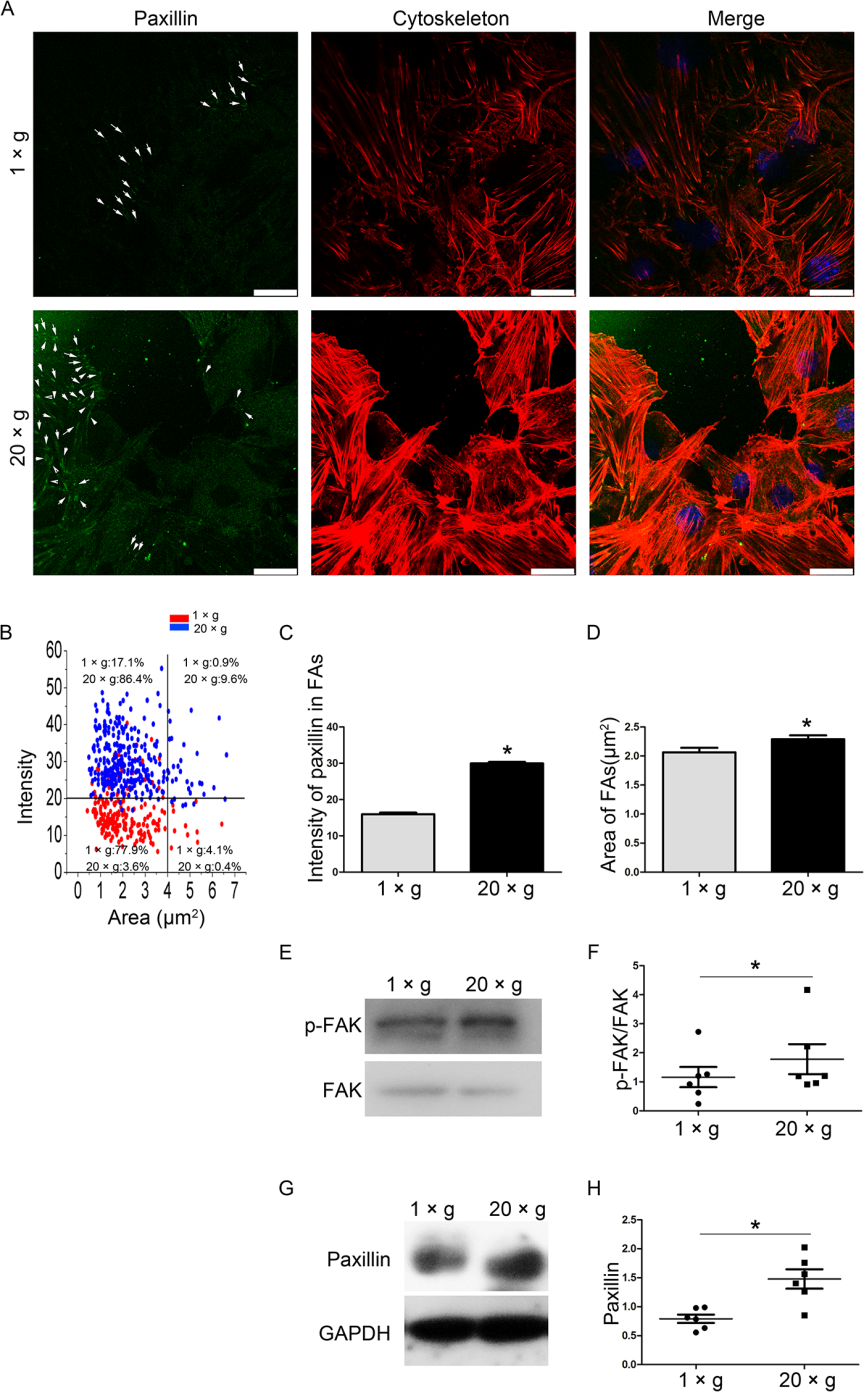
The effects of hypergravity on focal adhesions. (A) FITC (green) labeled paxillin in cells, and rhodamine phalloidin (red) labeled actin filaments. Focal adhesions are indicated by arrows (Scale bar = 25 μm). (B) The FAs in cells with or without hypergravity treatment mapped to the 2D diagram coordinate of average size and intensity. (C) The mean fluorescence intensity of paxillin in focal adhesions. ~150 focal adhesions were measured (mean ± SEM). (D) The mean size of focal adhesions. ~150 focal adhesions were measured (mean ± SEM). (E and F) Western blotting results of p-FAK/FAK expression in cells and statistical analyses (n = 6). (G and H) Western blotting results of paxillin expression in cells and statistical analyses (n = 6). n.s. indicates no statistical difference between 1 ×*g* and 20 ×*g*. *, statistically significant difference (*P* < 0.05).

Upon visualization of FAs with a paxillin antibody, we observed the fluorescence intensity, size, and number of FAs under normal gravity and at 20 ×*g* hypergravity. The number of observable FAs in the hypergravity-treated cells and the fluorescence intensity of each FA were higher than those in the unloaded cells (Fig 7A and 7C), while only a small difference in FA size was observed between unloaded (2.1 μm^2^) and post-hypergravity (2.3 μm^2^) samples (Fig 7D). When mapping the 2D diagram coordinates for average size and intensity of FAs, more FAs in the hypergravity-treated cells fell into the high-FA intensity and/or size region, when compared with those in the unloaded cells (Fig 7B). p-FAK and paxillin, two important proteins in focal adhesions, were assayed to evaluate FA assembly. Both p-FAK and paxillin expression were upregulated after hypergravity treatment (n = 6, p<0.05) (Fig 7E–7H). These results show that hypergravity strengthens actin fibers and focal adhesions in osteoblasts.

### Inhibition of actomyosin contractility diminishes the hypergravity effects on focal adhesions and OPN expression

Actin bundles, bearing contractions generated by actomyosin sliding, are tracks that propagate forces, and play crucial roles in mechanosensory signal transduction. FAs consist of several signaling molecules (including Src, Cas, Fak, Vinculin, and Integrin) that can undergo tension-dependent conformational changes to affect kinase activity, phosphorylation site availability, intracellular localization, and/or ligand affinity. Actin bundles, FAs, and their interactions play pivotal roles in several mechanosensory pathways [40–42]. Therefore, we proposed that actomyosin contraction of actin bundles and FAs may contribute to hypergravity-regulated Runx2 and OPN expression, and also determined the mechanosensing element that is the furthest upstream.

**Fig 8:**
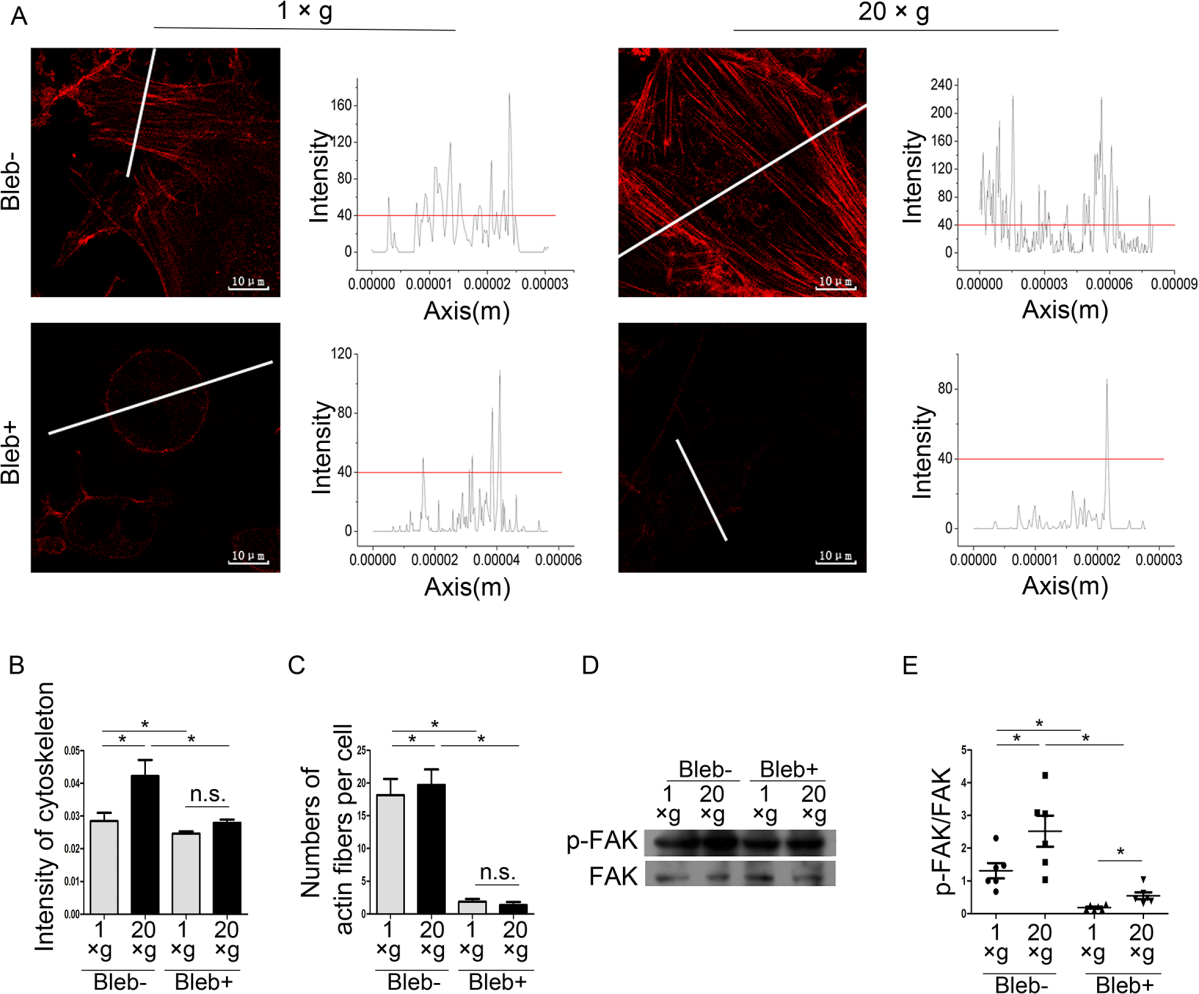
Blebbistatin inhibited hypergravity-induced effects on the cytoskeleton and focal adhesions. (A) After treated with blebbistatin, non-cortical actin alignment disappeared (Scale bar = 10 μm). (B) Blebbistatin inhibited the hypergravity-induced increase in the mean fluorescence intensity of actin fibers. ~250 cells were counted (mean ± SEM). (C) Blebbistatin treatment reduced the numbers of actin fibers in cells. ~50 cells were counted (mean ± SEM). (D) Western blotting results of p-FAK/FAK after blebbistatin treatments. (E) Statistical analyses of results in D (n = 6). n.s. indicates no statistical difference between 1 ×*g* and 20 ×*g*. *, statistically significant difference (*P* < 0.05). Blebbistatin (Bleb).

To explore the effects of actomyosin contraction on hypergravity-triggered signals, we used 50 μM blebbistatin to inhibit actomyosin contraction and assayed FA assembly, Runx2, and OPN expression. Immunofluorescence staining showed that non-cortical actin alignment and the differences in F-actin intensity between 20 ×*g*- and 1 ×*g*-treated cells disappeared after the blebbistatin treatment (Fig 8A and 8B). The number of actin fibers in each cell considerably decreased after blebbistatin treatment (Fig 8C). Western blotting showed that blebbistatin treatment failed to diminish hypergravity-induced FAK phosphorylation (Fig 8D and 8E).

**Fig 9:**
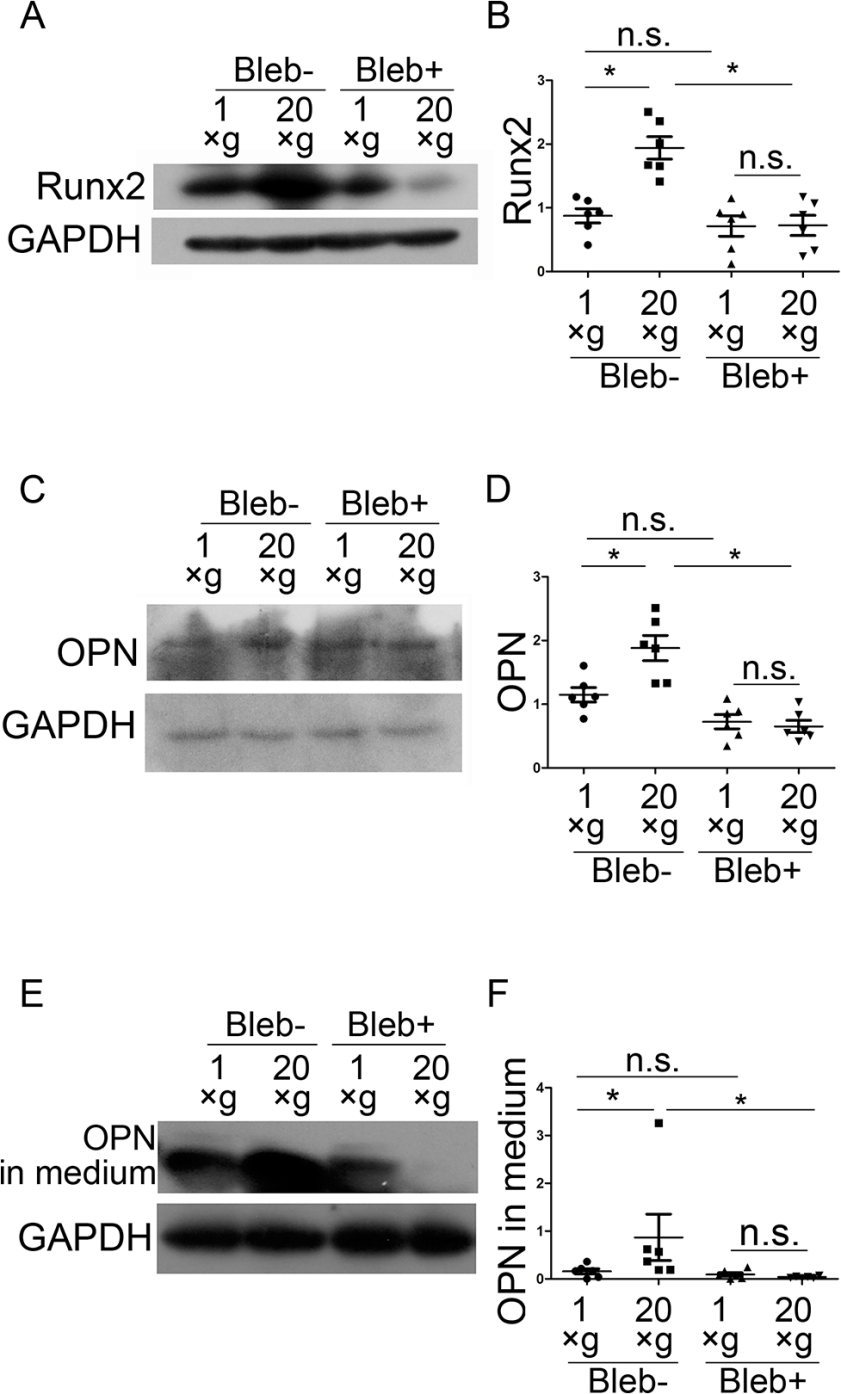
Blebbistatin inhibited hypergravity-induced OPN and Runx2 expression. (A and B) Western blotting results and statistical analyses of Runx2 expression in osteoblasts (n = 6). (C and D) Western blotting results and statistical analyses of OPN expression in cells (n = 6). (E and F) Western blotting results of OPN in culture medium and statistical analyses (n = 6). n.s. indicates no statistical difference between 1 ×*g* and 20 ×*g*. *, statistically significant difference (*P* < 0.05). Blebbistatin (Bleb).

Next, we examined Runx2 and OPN protein expression after blebbistatin treatment. Western blotting showed that blebbistatin treatment inhibited hypergravity-induced Runx2 and OPN expression, and OPN secretion (Fig 9). These results support our hypothesis that hypergravity affects OPN expression and secretion via regulation of actin bundles.

### FAs are involved in promoting hypergravity-induced OPN expression

**Fig 10:**
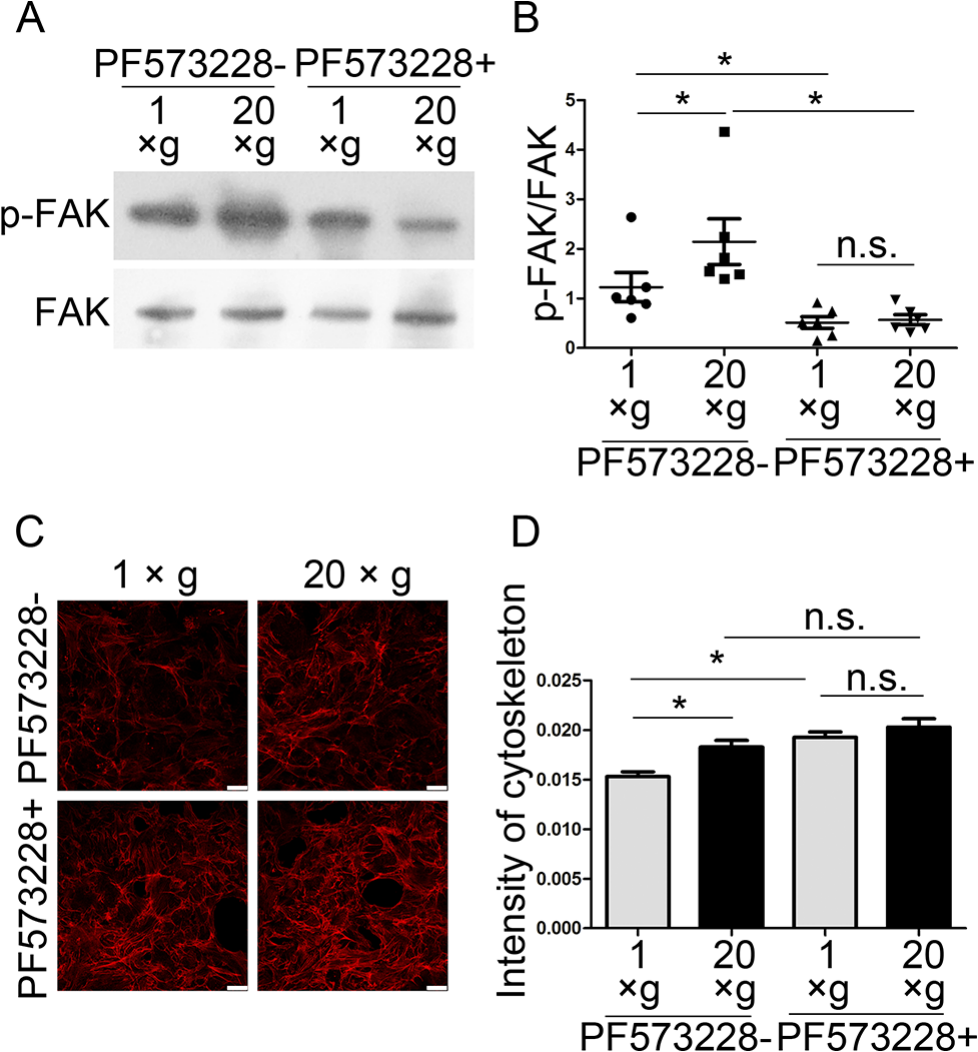
Effects of hypergravity on actin bundle intensity were inhibited by a FAK phosphorylation inhibitor. (A and B) Western blotting results showed that PF573228 inhibited FAK phosphorylation (n = 6). (C and D) Actin bundle intensity before and after PF573228 treatment and statistical analyses. ~250 cells were counted (mean ± SEM) (Scale bar = 25 μm). n.s. indicates no statistical difference between 1 ×*g* and 20 ×*g*. *, statistically significant difference (*P* < 0.05).

**Fig 11:**
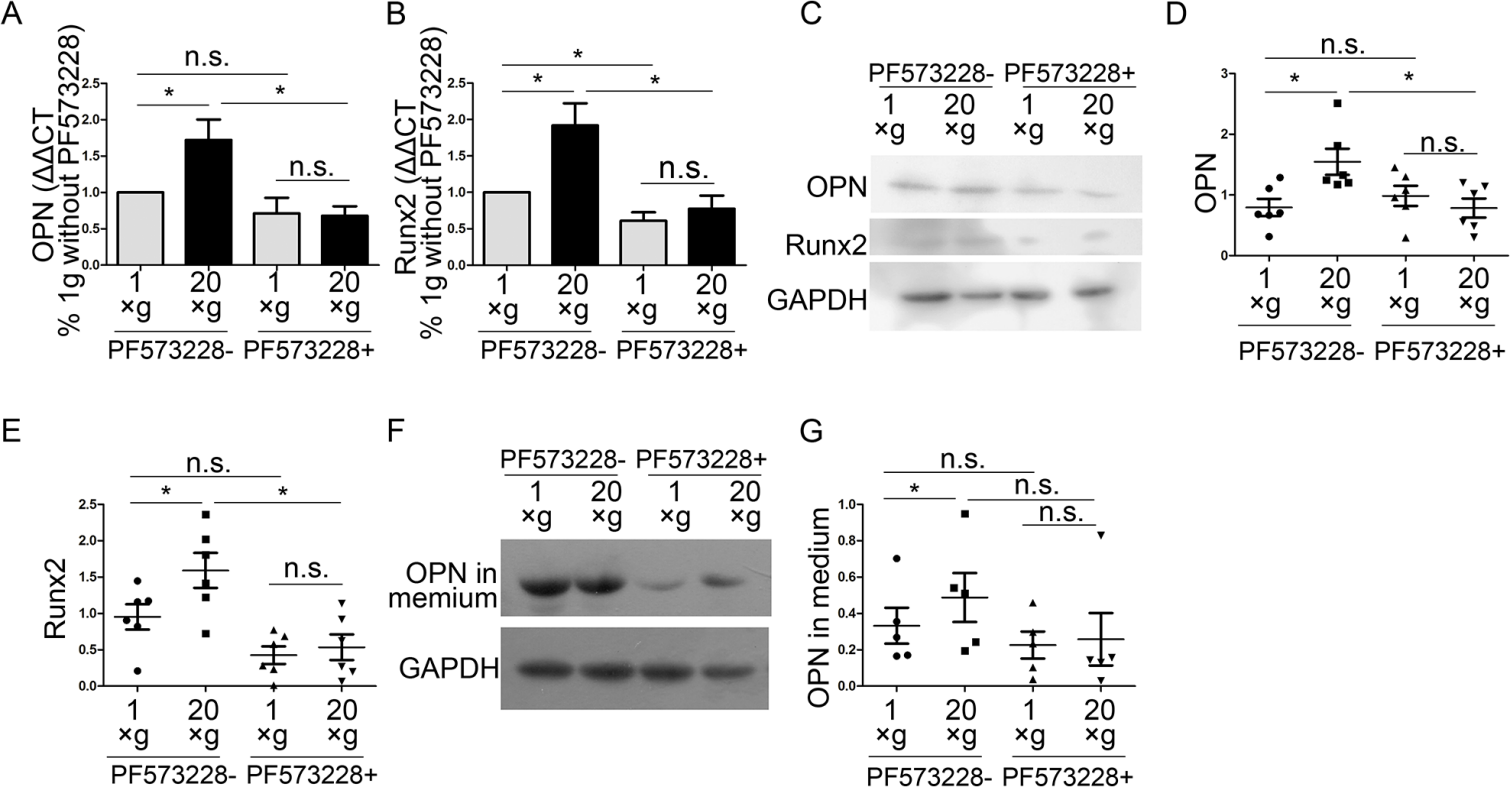
Effects of hypergravity on OPN and Runx2 expression were inhibited by a FAK phosphorylation inhibitor. (A and B) Real-time PCR results showed the expression of OPN and Runx2 (mean ± SEM; n = 4). (C) Western blotting showed that PF573228 inhibited the hypergravity-promoted OPN and Runx2 expression. (D and E) Statistical analyses of C (n = 6). (F and G) Western blotting of OPN in the culture medium and statistical analyses (n = 5). n.s. indicates no statistical difference between 1 ×*g* and 20 ×*g*. *, statistically significant difference (*P* < 0.05).

To investigate the role of FAs in hypergravity-induced OPN up-regulation, we used 1 μM PF573228 to inhibit FAK phosphorylation and then analyzed the cytoskeleton, and the expression of Runx2 and OPN. We observed that PF573228 inhibited FAK phosphorylation and hypergravity-induced p-FAK expression (Fig 10A and 10B). Fluorescence staining demonstrated that PF573228 treatment diminished the intensity differences in actin bundles between hypergravity-treated and unloaded cells (Fig 10C and 10D). Both the mRNA and protein levels of Runx2 and OPN were inhibited by PF573228 and no differences were observed in Runx2 and OPN expression between hypergravity-treated and unloaded cells (Fig 11). Since blebbistatin treatments failed to ablate hypergravity-promoted p-FAK expression, it is likely that FAK phosphorylation and actin bundles are involved in the hypergravity-induced increase in Runx2 and OPN expression, and also that FAK phosphorylation is upstream of actin bundle assembly.

## Discussion

In this study, we exposed osteoblasts to 20 ×*g* hypergravity by using a low-speed centrifuge for 24 h, and observed that hypergravity promoted OPN expression by facilitating FAs, actin bundle assembly, and Runx2 expression.

OPN expression is reported to be sensitive to altered gravity in space flight [43]. Our result, which showed that hypergravity can increase OPN expression in osteoblasts, supported the results of previous studies using other cell types, where short periods (60 min) of hypergravity stimulation increased OPN expression [44]. Here, we found that a medium-magnitude (20 ×*g*), 24 h continuous hypergravity stimulation upregulated OPN expression, and the hypergravity-increased OPN expression can last for at least 3 h after hypergravity deprivation. After 48 h hypergravity treatment, the expression of OPN and Runx2 returned to the same levels of the control group. Independent group has reported that OPN plays a role in the early the initial early molecular events during the course of bone and tendon remodeling [45, 46]. The present work indicates that OPN is involved in the early response of osteoblast cells to hypergravity. The mechanism underlying the dynamically regulated expression of OPN is unknown and warrants further investigation.

Some transcription factors such as AP-1 and ERK1 are known to bind the OPN promoter and affect OPN expression in certain cell types under hypergravity conditions [44]. In this study, we observed that Runx2 was a necessary element in facilitating the hypergravity-regulated OPN expression pathway. Runx2 can regulate OPN expression by binding to the OPN promoter [15]; therefore, hypergravity may affect OPN via Runx2.

FAs and the cytoskeletal system, including actin bundles, microtubules, and intermediate filaments, have long been known to respond to hypergravity and microgravity [39, 47,48]. Since proteins in FAs and actin bundles are well-documented mechanosensing/transducing elements, one may easily deduce that actin bundles and FAs may play important roles in the mechanotransduction pathway in cells under hypergravity. However, there are interactions and feedback pathways between FAs and actin bundle assembly, whose roles in the hypergravity-sensing pathway have not been fully elucidated. In the present study, using a multi-index analysis including the intensity, width, and distribution of actin bundles, we found that actomyosin contractility was important for hypergravity-induced upregulation of OPN and Runx2 expression, and that it was not responsible for FAK phosphorylation induced by hypergravity. The inhibition of p-FAK diminished actin bundle assembly, as well as OPN and Runx2 upregulation under hypergravity conditions. These results indicated that both FAK phosphorylation and actomyosin contraction play roles in hypergravity-induced OPN expression, and suggested that FAK is upstream of actin assembly.u

Since actin bundles are crucial to the cell’s reaction to hypergravity, the signaling pathways interacting with actin bundles, such as Rho/ROCK, ERK/ERK1, and ERK-c-Fos, are worth investigating [23,44]. In addition, the effects of other cytoskeletal components, microtubules, and intermediate filaments on cells subjected to hypergravity warrant further exploration. Notably, the response of the cytoskeletal system to hypergravity may depend on the specific cell type and the dose of hypergravity. Rat bone marrow mesenchymal stem cells cultured on a smooth surface have been reported to have a nonsignificant cytoskeletal response to 10 ×*g* hypergravity treatments [49]. Here, we also demonstrated that the cell density, the time length for the hypergravity treatment and the preparatory adherent period before hypergravity treatment have effect on the expression of OPN and Runx2. Thus, the effects of hypergravity on cell function and the underlying mechanisms should be carefully evaluated in seeding cells used for tissue engineering.

In conclusion, our findings show that a 24-h medium hypergravity treatment can upregulate OPN expression and secretion in osteoblasts. Furthermore, FAK phosphorylation, actin bundle contractility, and Runx2 are closely involved in the hypergravity sensing and signal transduction process. These results suggest that hypergravity conditions induced by centrifugation may be a convenient approach in bone tissue engineering.
